# Identification and functional characterization of SlDronc in *Spodoptera littoralis*

**DOI:** 10.7717/peerj.10329

**Published:** 2020-11-03

**Authors:** Hao Liu, Ke Zhou, Zhouning Yang

**Affiliations:** State Key Laboratory of Virology, Modern Virology Research Center, College of Life Sciences, Wuhan University, Wuhan, China

**Keywords:** Apoptosis, *Spodoptera littoralis*, SlDronc, SlIAP

## Abstract

**Background:**

Apoptosis is responsible for eliminating damaged and virus-infected cells, regulating normal cell turnover, and maintaining the immune system’s development and function. Caspases play a vital role in both mammal and invertebrate apoptosis. *Spodoptera littoralis* is a generalist insect herbivore that is one of the most destructive pests in tropical and subtropical areas and attacks a wide range of commercially important crops. Although *S. littoralis* is a model organism in the study of baculovirus infection, its apoptotic pathway has not been explored.

**Methods:**

We cloned a new caspase gene named *sldronc* in *S. littoralis* using Rapid Amplification of cDNA Ends (RACE). We then measured caspase activity on synthetic caspase substrates and *S. littoralis*’ effector caspase. SlDronc’s function in the apoptotic pathway and its interaction with caspase inhibitors were also tested in SL2 cells.

**Results:**

We found that the initiator caspase SlDronc cleaved and activated effector caspase in *S. littoralis*. SlDronc overexpression induced apoptosis in SL2 cells, and *Sldronc* knockdown decreased apoptosis induced by UV irradiation in SL2 cells. Our results indicate that SlDronc acts as an apoptotic initiator caspase in *S. littoralis*. Additionally, we found that processed forms of SlDronc increased in the presence of N-terminally truncated *S. littoralis* inhibitors of apoptosis (SlIAP) and that SlDronc was inhibited by P49. This study contributes to the further understanding of *S. littoralis’* apoptotic pathway and may facilitate future studies on baculovirus infection-induced apoptosis.

## Introduction

Apoptosis is a form of strictly controlled and programmed cell death that eliminates impaired and virus-infected cells (Clem, 2001; Duprez et al., 2009). Apoptosis plays a vital role in normal cell turnover and the proper development and function of the immune system (Elmore, 2007). Apoptosis mainly proceeds through the intrinsic and extrinsic pathways, both of which converge during caspase activation (Degterev, Boyce & Yuan, 2003; Hay & Guo, 2006; Tait & Green, 2010). Caspases are cysteine-dependent, aspartate-directed proteases that cleave numerous specific target sites in cellular proteins after upstream signal activation (Wallach et al., 2016). According to their localization in the apoptotic pathway and biological functions, caspases can be divided into several groups, including initiator caspases, effector caspases, inflammatory caspases, and caspases without a known function (Cohen, 1997; Galluzzi et al., 2016; Li & Yuan, 2008).

Caspases are synthesized as inactive zymogens, or pro-caspases. Pro-caspases have a prodomain in the N-terminal and a conserved catalytic domain in the C-terminal, which consists of a large subunit and a small subunit. During apoptosis, the pro-caspase is cleaved between the prodomain and large subunit, the cleavage between the large subunit and the small subunit is not essential for caspase activation. A heterodimer is made up of a large subunit and a small subunit, and an active unit of tetramer is formed by two heterodimers. Initiator caspase generally has a long prodomain containing caspase recruit domains (CARDs) or death effector domains (DEDs). CARDs and DEDs can combine with adapter proteins located in the upstream of initiator caspase in the apoptotic pathway via their homologous motifs. However, effector caspase has a short prodomain without a DED or CARD (Lo et al., 2015; Salvesen & Abrams, 2004). Initiator caspase is activated by dimerization that is facilitated by recruiting caspases to oligomeric activation platforms that assemble following an apoptotic signal (Donepudi et al., 2003; Dorstyn & Kumar, 2008; Pop et al., 2006). The activated initiator caspase activates effector caspase through proteolytic cleavage (Pop & Salvesen, 2009).

There are seven caspases in *Drosophila melanogaster*, a model organism in the study of insect apoptosis. Dronc and Dredd are initiator caspases with prodomains that have one CARD or two DEDs, respectively; Drice, Dcp-1, Decay, and Damm are effector caspases (Cooper, Granville & Lowenberger, 2009; Kumar & Doumanis, 2000). Dronc acts as an upstream caspase in the intrinsic pathway and its activation mechanism resembles that of mammalian caspase-9 (Dorstyn et al., 1999; Muro, Monser & Clem, 2004). Lepidopteran caspases are classified into six clades. Lep-Caspase-1, Lep-Caspase-2, and Lep-Caspase-3 are the supposed effector caspases, and Lep-Caspase-5 and Lep-Caspase-6 are the supposed initiator caspases. Dronc belongs to the Lep-caspase-5 clade (Courtiade et al., 2011). Previous studies have reported on Dronc homologs from several Lepidopteran insects, including *Bombyx mori* (BmDronc), *Lymantria dispar* (LdDronc), and *Spodoptera frugiperda* (SfDronc) (Huang et al., 2013; Kitaguchi et al., 2013; Suganuma et al., 2011).

Apoptosis is regulated by multiple cellular proteins, including inhibitor of apoptosis (IAP) which function as the last line of defense against caspase-mediated apoptosis. IAP can inhibit caspases by directly binding to them through baculoviral IAP repeat (BIR) domains or ubiquitylating caspases with the RING domain following binding (Deveraux et al., 1997; Ditzel et al., 2008; Roy et al., 1997). IAP need to be processed by caspases in order to work. For example, DIAP1 requires caspase-mediated cleavage to function, drICE cleaves 20 N-terminal amino acids to activate DIAP1’s ability to suppress apoptosis, and DIAP1’s C-terminal is degraded by the N-end rule degradation pathway (Ditzel et al., 2003; Yan et al., 2004). The N-end rule pathway is a proteolytic system that depends on proteasome, and recognizes and degrades proteins that have N-degrons (Gibbs et al., 2014; Tasaki et al., 2012).

Apoptosis is also regulated by inhibitors in baculovirus. P35 in *Autographa californica* multiple nucleopolyhedrovirus (AcMNPV) and P49 in *Spodoptera littoralis* nucleopolyhedrovirus (SpliNPV) are two baculoviral apoptosis inhibitors. Generally, baculoviral apoptosis inhibitor P49 inhibits the caspase activity of initiator caspases and baculoviral apoptosis inhibitor P35 inhibits the caspase activity of effector caspases (Jabbour et al., 2002; Zoog et al., 2002).

*S. littoralis* is a generalist insect herbivore that targets a wide range of commercially important crops, including cotton, rice, maize, and potato (Lee & Anstee, 1995). *S. littoralis* is distributed across Africa, the Mediterranean region, and the Near East, and is one of the most destructive pests in tropical and subtropical areas (Hill, 1987). SL2 cells derived from *S. littoralis* and Sf9 cells derived from *S. frugiperda* are often used when studying baculovirus infection and apoptosis (Mialhe, Quiot & Paradis, 1984). Compared to Sf9 cells, SL2 cells undergo apoptosis and produce very low levels of polyhedrin when infected with AcMNPV (Chejanovsky & Gershburg, 1995), suggesting that SL2 and Sf9 cells have different apoptosis mechanisms. However, several years have passed since the first study on effector caspase, Sl-caspase-1, and *S. littoralis* inhibitors of apoptosis (SlIAP) was published (Liu, Qi & Chejanovsky, 2005; Vilaplana et al., 2007). Since then, no initiator caspases have been identified and very few articles have expounded on the apoptosis mechanism of *S. littoralis*. In this study, we identified SlDronc, an initiator caspase in *S. littoralis*. We analyzed the amino acid sequences of SlDronc, tested its biochemical character, and verified its function in SL2 cells. This study contributes to the further understanding of *S. littoralis’* apoptotic pathway and may facilitate future research on baculovirus infection-induced apoptosis.

## Materials & Methods

### Cells

SL2 cells were kindly gifted by Professor Nor Chejanovsky, Agricultural Research Organization, Volcani Center, Israel. SL2 cells were cultured using Grace’s insect medium (Invitrogen, Carlsbad, CA, USA) at 27 °C in a biochemical incubator, and 10% (v/v) heat-inactivated fetal bovine serum (FBS, Gibco, Waltham, MA, USA) was added to the insect medium.

### Antibodies

Rat-derived monoclonal antibodies against His-tag, Flag-tag, HA-tag, and β-actin (Proteintech, Rosemont, IL, USA) were diluted in block buffer (1:5000) to be used for Western blot analysis. We diluted rabbit-derived polyclonal antibody against Sf-caspase-1 (also provided by Professor Nor Chejanovsky), which can recognize full-length, large subunits of Sf-caspase-1 and Sl-caspase-1, 1:1000 in block buffer to be used for western blotting. A polyclonal antibody against SlDronc, which can recognize full-length and large subunits of SlDronc, was produced using a SlDronc fragment purified in *E. coli* as an antigen to immunize rabbits. We produced a polyclonal antibody against SfIAP, which can also recognize full-length, cleaved SlIAP, using a SfIAP fragment purified in *E. coli* as an antigen to immunize rabbits.

### Cloning *S. littoralis dronc*

We first cloned *Sldronc* as a partial sequence using the designed primers according to the alignment of *dronc* homologs from *S. frugiperda* (*Sfdronc*) and *Spodoptera litura* (*Sl-caspase-5*). The sequence contained an intact open reading frame (ORF) that was highly similar to *Sfdronc*’s. To determine *Sldronc*’s full length, we designed primers for Rapid Amplification of cDNA Ends (RACE) according to *Sldronc*’s obtained partial sequence (Table 1). We used the SMARTer™ RACE cDNA Amplification Kit (Clontech, Mountain View, CA, USA) in the 5′ and 3′ RACE to amplify *Sldronc*’s coding sequence and untranslated regions (UTR). The purified PCR products were ligated into the pCR-II vector (TA Cloning^®^ Kit, Invitrogen, Carlsbad, CA, USA) and plasmids extracted from several positive colonies were sequenced. Our sequencing results showed an assembled sequence containing a 1,338-bp ORF with a 216-bp 5′ UTR and a 397-bp 3′ UTR . We named this ORF *Sldronc* and cloned it into the pCR-II vector from *S. littoralis* cDNA. Plasmids extracted from several positive colonies were sequenced, confirming the sequence information from RACE.

**Table 1:** Primers used for RACE of SlDronc.

Primer name	Primer sequence
1-17-5′GSP1	CCTCTTATTGAAGCCCCAGCGGTCCAC
1-17-5′NGSP1	GTATGTCGGAGTAGAGCGGCGTCTGTC
1-17-3′GSP1	GATTCCACCCCATTCCCCAACCTAAGC
1-17-3′NGSP1	GATGAACTCGGCTTTCGTAGGCTCGG

### Constructing plasmids

We constructed plasmid pET28a-SlDronc-C-His-expressing SlDronc with a His-tag at the C-terminal by ligating SlDronc’s ORF into Nco I and Hind III sites in vector pET-28a. The plasmids pET28a-Sl-caspase-1-C-His and pET28a-P49-(GS)_3_-C-His were used for Sl-caspase-1 and P49 expression containing a C-terminal His-tag in *E. coli*. Plasmid pIE1-SlDronc-C-Flag was used for SlDronc overexpression containing a C-terminal Flag-tag in SL2 cells. Plasmid pIE1-N-HA-SlDronc was used for SlDronc overexpression containing an N-terminal HA-tag in SL2 cells. Plasmid pIE1-P49-C-His was used for P49 overexpression with a C-terminal His-tag in SL2 cells. Plasmid pIE1-SlIAP-C-Flag was used for SlIAP overexpression with a C-terminal Flag-tag in SL2 cells. We constructed SlDronc C310A, Sl-caspase-1 C178A, SlIAP D87A, and SlIAP N88G by introducing point mutation into wild type plasmids using site-directed mutagenesis PCR. The forward and reverse primers are listed in Table 2. DNA sequencing was used to verify all constructed plasmids.

**Table 2:** Primers used for mutagenesis.

Primer name	Primer sequence
SlDronc C310A(S)	CAAATGGCCAGAGGCAGCAGTG
SlDronc C310A(A)	CTGCTGCCTCTGGCCATTTGG
Sl-caspase-1 C178A(S)	GCTGCCCAAGGTGATAAATTG
Sl-caspase-1 C178A(A)	CTTGGGCAGCTTGAATAAAG
SlIAP D87A-AS	GTCGTGATTGGCGGTTTTATCGATG
SlIAP D87A-S	GATAAAACCGCCAATCACGACACC
SlIAP N88G-AS	GTCGTGACCGTCGGTTTTATCGATG
SlIAP N88G-S	GATAAAACCGACGGTCACGACACC

### Purifying recombinant proteins

We cultured *E. coli* BL21 (DE3)/pLysS cells transfected by plasmid expressing interest protein or vector in LB medium with 50 µg/mL kanamycin until it reached an OD_600nm_ concentration between 0.4 and 0.6. The interest protein expression was induced by 0.2 mM isopropyl-β-D-thiogalactopyranoside (IPTG) and the cells were cultured for another 2 h at 200 rpm. We used 20 mM imidazole solution with 1% detergent Triton X-100 and protease inhibitor (Roche, Basel, Switzerland) to resuspend BL21 cells after centrifugation, then sonicated the suspension for 4 s with 6 s intervals for a total of 30 min, and then 10-min intervals between every 10 min sonication. The BL21 cell lysate was centrifugated at 14,000 g for 30 min at 4 °C, and Ni-NTA high affinity resin (Genscript, Piscataway, NJ, USA) was incubated with the supernatant according to the manufacturer’s instructions. We used 20 mM, 50 mM, and 80 mM imidazole solution to wash off the resin and 250 mM imidazole solution to elute the interest proteins. After Western blotting and Coomassie staining analyses, we stored the purified interest proteins at −80  °C.

### SDS-PAGE and western blotting

SDS-PAGE samples were prepared by mixing purified proteins or cell lysates with 5 ×SDS loading buffer, and then heating the mixture in boiling water for 10 min. We detected proteins using Coomassie blue staining or western blotting on a PVDF membrane (Millipore, Burlington, MA, USA). We used 5% milk or BSA in TBST to block the membranes for 1 h at room temperature, diluted the primary antibodies in block buffer, and incubated the membranes for 1 h. After washing three times with TBST, we incubated the horseradish peroxidase (HRP)-conjugated secondary antibodies (Thermo Fisher, Waltham, MA, USA) that had been diluted in block buffer with the membranes for 1 h. LAS 4000 (Fujifilm, Tokyo, Japan) was used to detect the protein-primary antibody-secondary antibody-HRP complex after incubating the membranes with chemiluminescent substrate (Millipore).

### Caspase activity assay

In order to analyze the substrate preference of recombinant SlDronc purified in *E. coli*, we used 13 kinds of fluorogenic synthetic caspase substrates: Ac-VEID-AFC, Ac-LETD-AFC, Ac-IETD-AFC, Ac-LEHD-AFC, Ac-AEVD-AFC, Ac-WEHD-AFC, Ac-DEVD-AFC, Ac-YVAD-AFC, Ac-DMQD-AFC, Ac-LEED-AFC, Ac-IEPD-AFC, Ac-VDVAD-AFC, and Ac-LEVD-AFC (MP). We also measured the effects of Sl-caspase-1 and the cell lysates on Ac-DEVD-AFC. We mixed purified caspase, incubate, or cell lysate with the appropriate fluorogenic substrate in Na-Citrate buffer (1 M Na-Citrate, 50 mM Tris-base, 10 mM DTT, 0.05% CHAPS, pH 7.4), and used 20 µM substrate in 100 µL of each test mixture (Pop, Salvesen & Scott, 2008). The relative fluorescence unit was detected at 37  °C every 2 min for 2 h after 30 min of incubation at 37 °C. We used the data to calculate the maximum slope of each curve and generated the graph using GraphPad Prism 6.

### Transfection

SL2 cells were transfected using the method previously described (Summers & Smith, 1987). SL2 cells were seeded into a culture plate at a cell abundance of about 70%. After the attachment period, we removed the medium and added a mixture of plasmid, transfection buffer (25 mM HEPES, pH 7.1, 140 mM NaCl, and 125 mM CaCl_2_), and Grace’s insect medium dropwise into the well with the SL2 cells. The cells were maintained at 27 °C for 4 h before we replaced the transfection mixture with fresh Grace’s medium. We used 3.0 µg plasmid per well in the 12-well plate.

### UV irradiation

We treated the SL2 cells with UV irradiation by placing the cell culture plate on a transilluminator for 45 min. Twenty-four hours after UV treatment, the cells were harvested and lysed for further analysis.

### Cell lysate preparation

SL2 cells were harvested via centrifugation of the culture medium at 1,000 g for 10 min. Cell lysates were prepared using the following procedure. Lysis buffer (200 mM Tris–HCl pH 7.4, 150 mM NaCl, 1 mM EDTA, and 1% NP-40) mixed with protease inhibitor (Roche) was used to suspend the cells (Yang et al., 2016). After three freeze-thaw cycles, we centrifuged the suspension at 14,000 g at 4 °C for 10 min, and then removed the supernatants as cell lysates for analysis. When harvesting the apoptotic SL2 cells, we first harvested cells at 3,000 g at 4 °C for 10 min, and then centrifuged the supernatant to collect the apoptotic bodies at 14,000 g at 4 °C for 20 min. The harvested cells and apoptotic bodies were gathered for cell lysate preparation using the routine method.

### Gene knockdown

We cloned the DNA fragments of interest using primers (Table 3) that contained T7 promoter in the 5′ end and plasmids or cDNAs that contained desire sequences as templates. RNAs were transcribed *in vitro* using the cloned DNA fragments. We removed the template DNA by adding TURBO DNase, and we extracted and purified the RNAs using phenol-chloroform. To obtain dsRNAs, the RNA products were slowly cooled to room temperature after incubation at 95 °C for 2 min. We used the NanoDrop One (Thermo Fisher) to determine dsRNA qualities and concentrations. We placed 1 µg dsRNA in each 6 × 10^5^ SL2 cell to knock down the desire gene. The dsRNA transcribed from the *gfp* gene was utilized as the negative control.

**Table 3:** Primers used for dsRNA.

Primer name	Primer sequence
T7-SlDronc-dsRNA-F	TAATACGACTCACTATAGGGCATCCAAGCATTGTGCGAGGT
T7-SlDronc-dsRNA-R T7-Sl-caspase-1-dsRNA-F T7-Sl-caspase-1-dsRNA-R	TAATACGACTCACTATAGGGTGACTATTACCTGCTACTCTGTTA TAATACGACTCACTATAGGGCCATTATTTTCAATCACGAGCATTT TAATACGACTCACTATAGGGCTAGAGCTACTTTCTGACACACA
T7-GFP-dsRNA-F T7-GFP-dsRNA-R	TAATACGACTCACTATAGGGATGGTGAGCAAGGGCGAGGA TAATACGACTCACTATAGGGTTGAAGTTCACCTTGATGCC

## Results

### SlDronc sequence analysis

**Figure 1:**
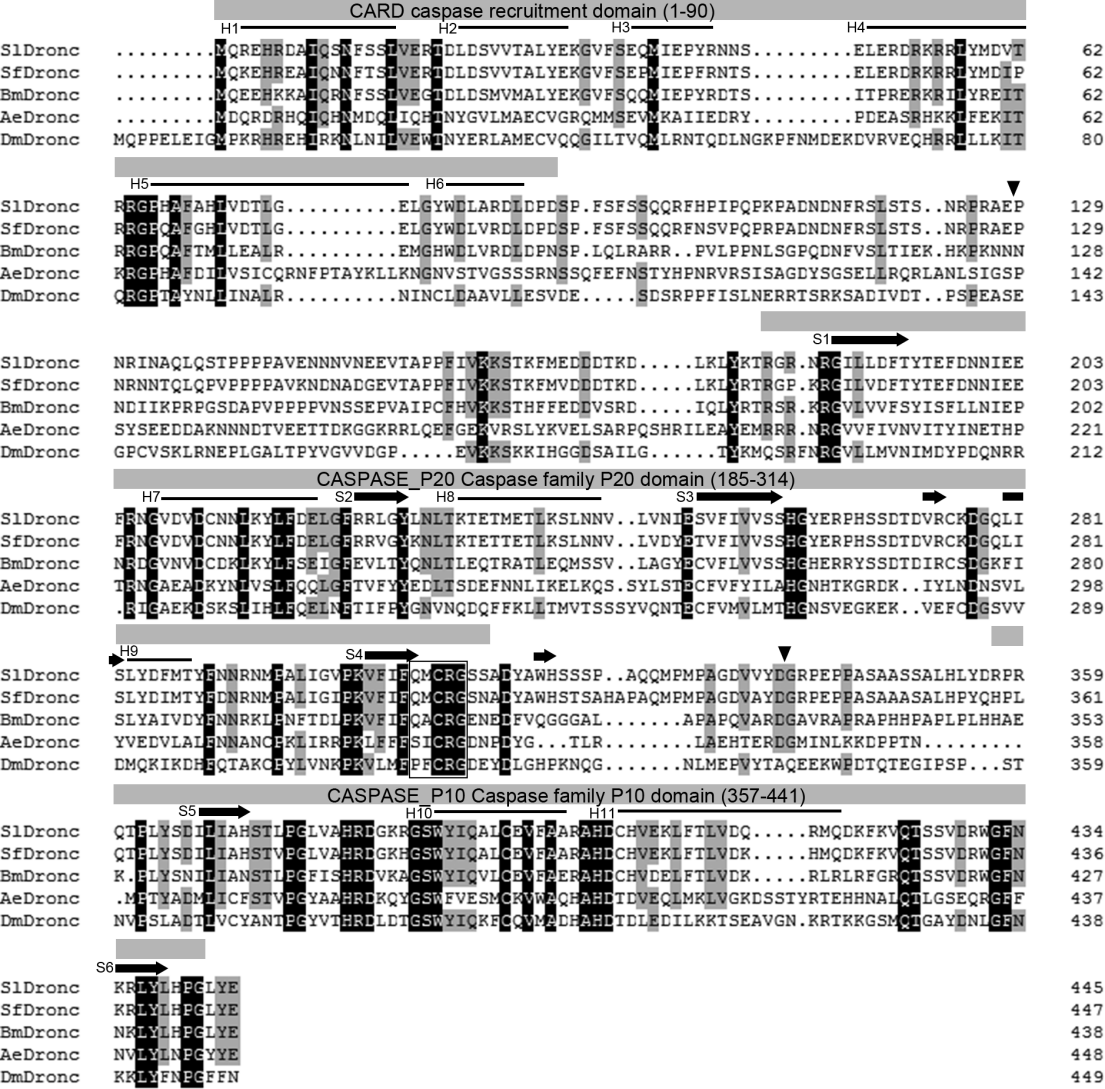
SlDronc sequence analysis. SlDronc amino acid sequence is shown aligned to Dronc homologs including SfDronc, BmDronc, AeDronc and DmDronc. Uniform amino acid residues in five Droncs are shown in white on a black background, the amino acid residues identical in four Droncs are shown in black on a shaded background. The CARD domain, caspase P20 domain, caspase P10 domain, predicted α-helices and β-sheets are marked above the sequence. A box is used to outline the predicted catalytic site, and inverted triangles are used to line out cleavage sites at position E128 and D338. The alignment was performed using DNAMAN Version 7. InterProScan Version 5.0 and PsiPred Version 3.3 were used to predict the conserved motifs and secondary structure in SlDronc.

We acquired a novel caspase cDNA, *Sldronc*, using RT-PCR on total mRNA from SL2 cells as a template. The predicted protein encoded by the *Sldronc* ORF contained 445 amino acids (about 51 kDa), which included a 15 kDa prodomain, a 24 kDa large subunit, and a 12 kDa small subunit. The alignment of SlDronc’s predicted amino acid sequence with Dronc homologs SfDronc, BmDronc, AeDronc, and DmDronc showed that SlDronc had 87%, 54%, 27%, and 24% consistency ratios with SfDronc, BmDronc, AeDronc, and DmDronc, respectively (Fig. 1). SlDronc also had a predicted typical caspase catalytic site sequence Q^308^MCRG^312^ (Fig. 1). The predicted secondary structure of SlDronc contained a series of α-helices and β-sheets that were highly conserved with Droncs in other insects (Watt et al., 1999; Fig. 1). SlDronc possesses a long prodomain containing a CARD, E128 is predicted to be a cleavage site between prodomain and large subunit, and D338 is predicted to be the cleavage site between the large and small subunit according to the verified cleavage sites in other Droncs (Fig. 1).

**Table 4:** Sequences used for alignments and phylogenetic tree.

Species	Name	Length	GenBank accession
*Aedes aegypti*	AeDredd	493 aa	ABI74776.1
*Aedes aegypti*	AeDronc	449 aa	XP_001655433.1
*Bombyx mori*	Bm-caspase-1	293 aa	NP_001037050.1
*Bombyx mori*	Bm-caspase-3/a (BmICE)	284 aa	ABC94941.1
*Bombyx mori*	Bm-caspase-4	497 aa	AEK71902.1
*Bombyx mori*	Bm-caspase-5 (BmDronc)	438 aa	NP_001182396.1
*Bombyx mori*	Bm-caspase-6 (BmDredd)	543 aa	BAF98475.1
*Drosophila melanogaster*	Dcp1	323 aa	NP_476974.1
*Drosophila melanogaster*	DECAY	308 aa	AAD54071.2
*Drosophila melanogaster*	DAMM	255 aa	AAF58613.3
*Drosophila melanogaster*	DrICE	339 aa	CAA72937.1
*Drosophila melanogaster*	STRICA (Dream)	527 aa	AAF57292.2
*Drosophila melanogaster*	DmDredd	494 aa	AAC33117.1
*Drosophila melanogaster*	DmDronc	450 aa	AAD26625.1
*Galleria mellonella*	Gm-caspase-6	537 aa	AEK20837.1
*Helicoverpa armigera*	Ha-caspase-5	453 aa	AEK20835.1
*Helicoverpa armigera*	Ha-caspase-6	542 aa	AEK20838.1
*Heliothis virescens*	Hv-caspase-6	547 aa	HQ328982.1
*Lymantria dispar*	Ld-caspase-5	443 aa	BAL60586.1
*Manduca sexta*	Ms-caspase-6	543 aa	AEF30497.1
*Pieris rapae*	Pr-caspase-5 (partial)	252 aa	AEK20836.1
*Spodoptera exigua*	Se-caspase-5	453 aa	AFX60235.1
*Spodoptera exigua*	Se-caspase-6	548 aa	AFO64608.1
*Spodoptera frugiperda*	Sf-caspase-1	299 aa	AAC47442.1
*Spodoptera frugiperda*	SfDronc	447 aa	JX912275
*Spodoptera frugiperda*	SfDredd	552 aa	AMR71144.1
*Spodoptera littoralis*	Sl-caspase-1	299 aa	AAO16241.1
*Spodoptera littoralis*	Sl-caspase-3	281 aa	AEK20824.1
*Spodoptera litura*	Spli-caspase-5	445 aa	AFJ04535.1
*Spodoptera litura*	Spli-caspase-6	522 aa	AFJ04536.1

### SlDronc phylogenetic analysis

**Figure 2:**
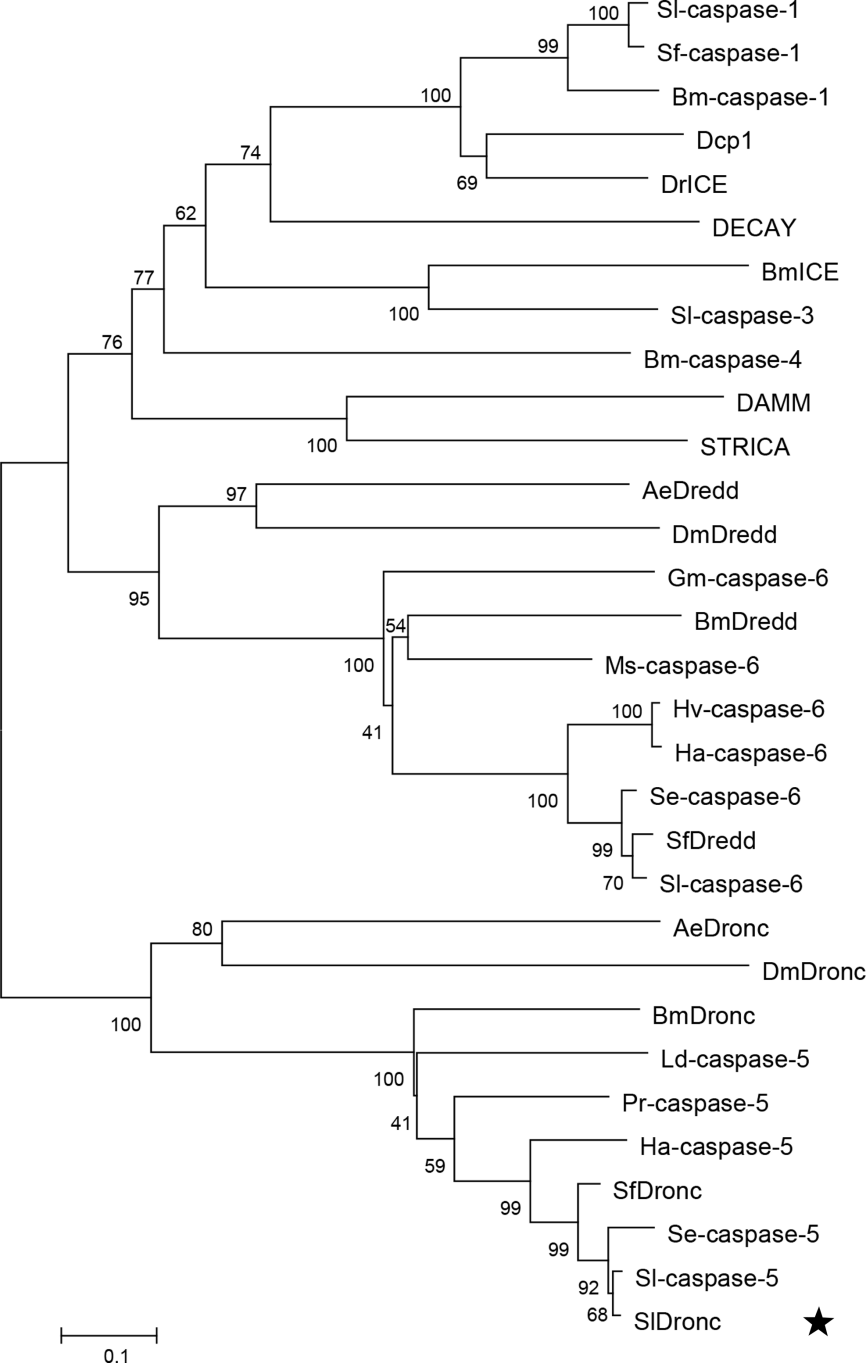
SlDronc phylogenetic analysis. Amino acid sequence of SlDronc and 30 chosen insect caspases were utilized to biuld the phylogenetic tree using the neighbor-joining method by MEGA Version 5.05. The sequences contain the following: SlDronc, Sl-caspase-1 and Sl-caspase-3 in *Spodoptera littoralis* , Spli-caspase-5 and Spli-caspase-6 in *Spodoptera litura*, Se-caspase-5 and Se-caspase-6 in *Spodoptera exigua*, Ha-caspase-5 and Ha-caspase-6 in *Helicoverpa armigera*, SfDredd, SfDronc and Sf-caspase-1 in *Spodoptera frugiperda*, Hv-caspase-6 in *Heliothis virescens*, Ms-caspase-6 in *Manduca sexta*, Gm-caspase-6 in *Galleria mellonella*, Bm-caspase-1, BmICE, Bm-caspase-4, BmDronc and BmDredd in *Bombyx mori*, Ld-caspase-5 in *Lymantria dispar* , Pr-caspase-5 in *Pieris rapae*, AeDronc and AeDredd in *Aedes aegypti*, Dcp1, DECAY, DAMM, DrICE, STRICA, DmDronc and DmDredd in *Drosophila melanogaster*. GenBank accession numbers of above sequences are listed in Table 4.

Our phylogenetic analysis of SlDronc and 30 chosen caspases in several insects (Table 4) suggested that the closest relatives of SlDronc belong to the Dronc homolog clade, they are Sl-caspase-5, Se-caspase-5, SfDronc, and Ha-caspase-5 in lepidopteran insects (Fig. 2).

### SlDronc autocatalytic cleavage in *E. coli*

**Figure 3:**
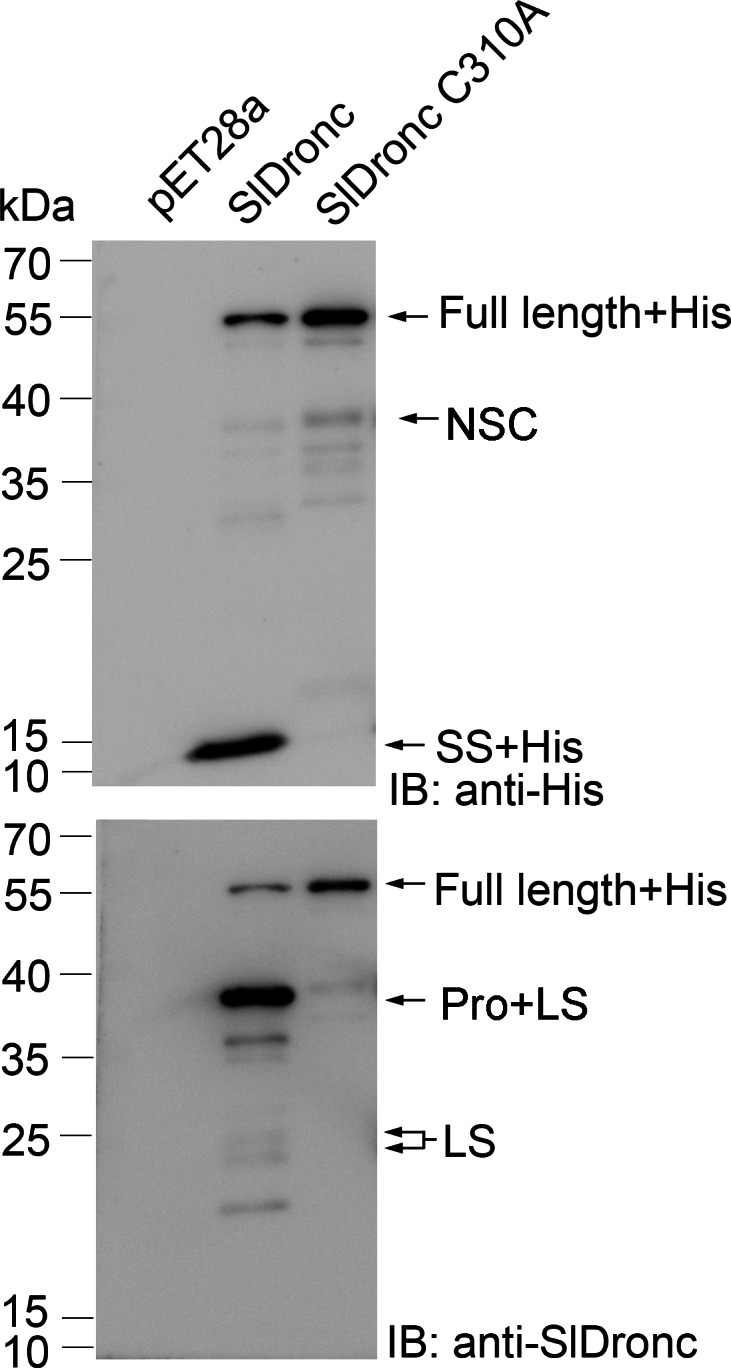
SlDronc autocatalytic cleavage in *E. coli*. Wild type SlDronc and the catalytic site mutant SlDronc C310A C-terminal His-tag were expressed in *E. coli* and purified proteins were detected by western blotting utilizing antibody against His-tag or SlDronc following SDS-PAGE. His represents His-tag , Pro represents prodomain, LS represents large subunit, SS represents small subunit. NSC represents Band might be due to non-specific cleavage by a bacterial protease.

Caspases usually autoprocess when brought into close proximity with each other according to the “induced-proximity” model (Riedl & Shi, 2004; Salvesen & Dixit, 1999). Consistent with this model, we detected three major bands (about 52 kDa, 38 kDa, and 13 kDa) by western blotting when recombinant SlDronc with C-terminal His-tag was expressed and purified in *E. coli* (Fig. 3). The 52-kDa band was predicted to be the full length of SlDronc plus the C-terminal His-tag (Full length+His). We predicted that the 38-kDa band was the fragment consisting of the prodomain and large subunit (Pro+LS). The 13-kDa band matched the supposed fragment consisting of the small subunit and the C-terminal His-tag (SS+His). The two forms of cleaved bands indicated that SlDronc underwent autocatalytic cleavage when expressed and purified in *E. coli*. In order to confirm whether the autocleavage of wild type SlDronc relies on its caspase activity, we mutated cysteine (C) at the 310 site to alanine (A) in SlDronc’s predicted catalytic site. Western blotting detected that the catalytic site mutant SlDronc C310A was synchronously expressed and purified in *E. coli* (Fig. 3). However, the SlDronc C310A did not autoprocess between the large subunit and small subunit, and we detected one major band that was supposed to be the full length of SlDronc in purified SlDronc C310A boring a C-terminal His-tag. These results indicate that SlDronc’s caspase activity was essential for autocatalytic cleavage and that SlDronc possessed caspase activity.

### Recombinant SlDronc’s strong activity on initiator caspase substrates

**Figure 4:**
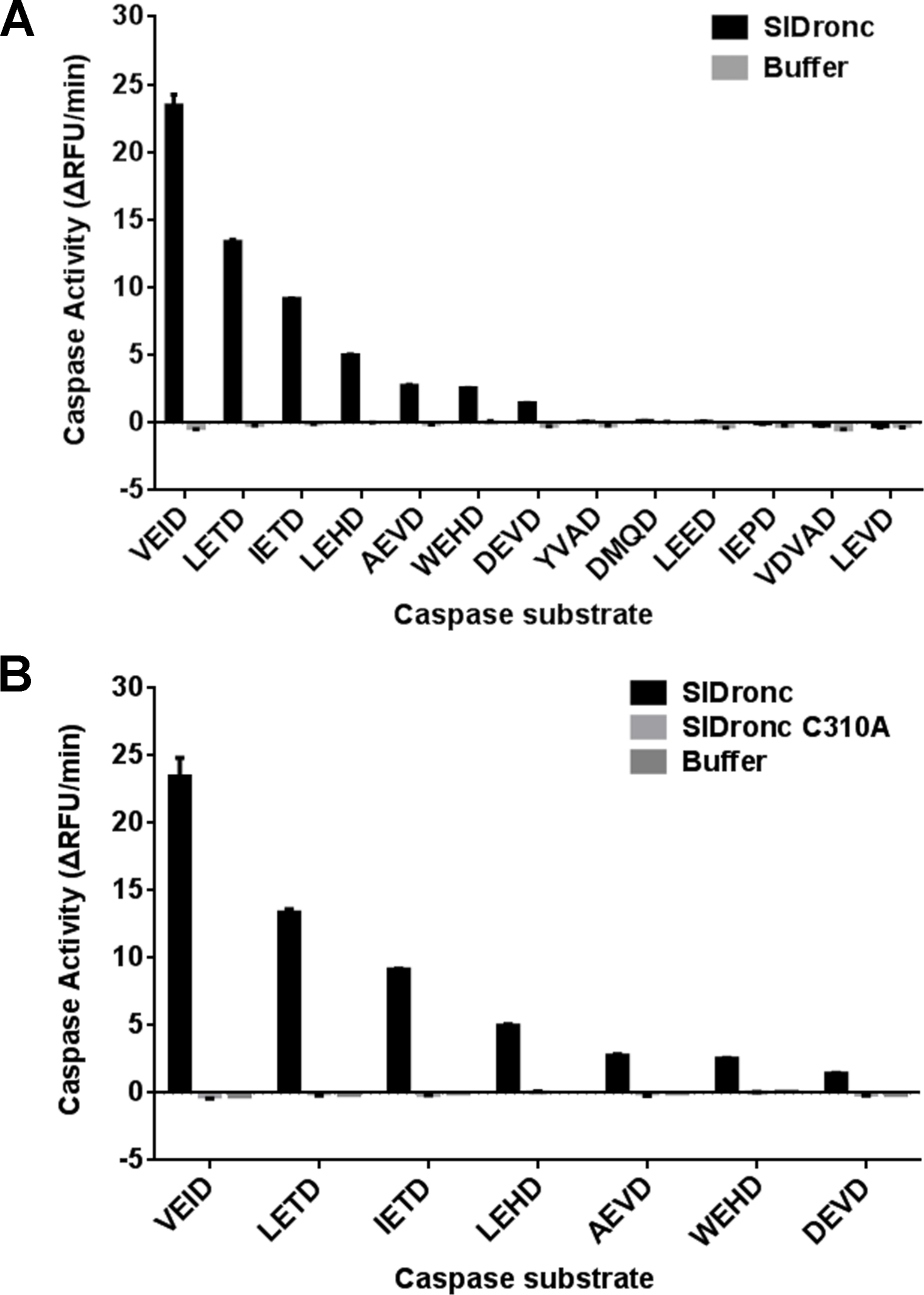
Recombinant SlDronc’s strong activity on initiator caspase substrates. (A) and (B) Wild type SlDronc (A) and catalytic site mutant SlDronc C310A (B) containing a His-tag in C-terminal were incubated with 13 kinds of synthetic caspase substrates or seven selected kinds of synthetic caspase substrates (20 µM) respectively, and then the mixture were subjected to caspase activity assay. Changes in RFU (relative fluorescence units) per minute was used to show caspase activity. SD from three independent experiments were presented.

Synthetic caspase substrates have been widely used to identify caspase activity (Pop, Salvesen & Scott, 2008; Talanian et al., 1997). To verify that SlDronc indeed has enzymatic activity, we measured the catalytic activities of recombinant SlDronc with a C-terminal His-tag on 13 kinds of caspase substrates. Comparing to the control, SlDronc with a C-terminal His-tag showed dramatic activities on Ac-VEID-AFC, Ac-LETD-AFC, Ac-IETD-AFC, Ac-LEHD-AFC, Ac-AEVD-AFC, Ac-WEHD-AFC, and Ac-DEVD-AFC (Fig. 4A), and SlDronc C310A with a C-terminal His-tag showed no activity on the selected substrates (Fig. 4B). Catalytic site mutation completely inhibited SlDronc activity, suggesting that wild type SlDronc activities on caspase substrates rely on caspase activity. This is consistent with the fact that SlDronc shows a high sequence similarity to initiator caspase, and SlDronc showed the strongest activity against Ac-VEID-AFC, a preferential substrate of initiator caspase. These data suggest that SlDronc may be an initiator caspase.

### SlDronc’s in vitro cleavage and activation of Sl-caspase-1

**Figure 5:**
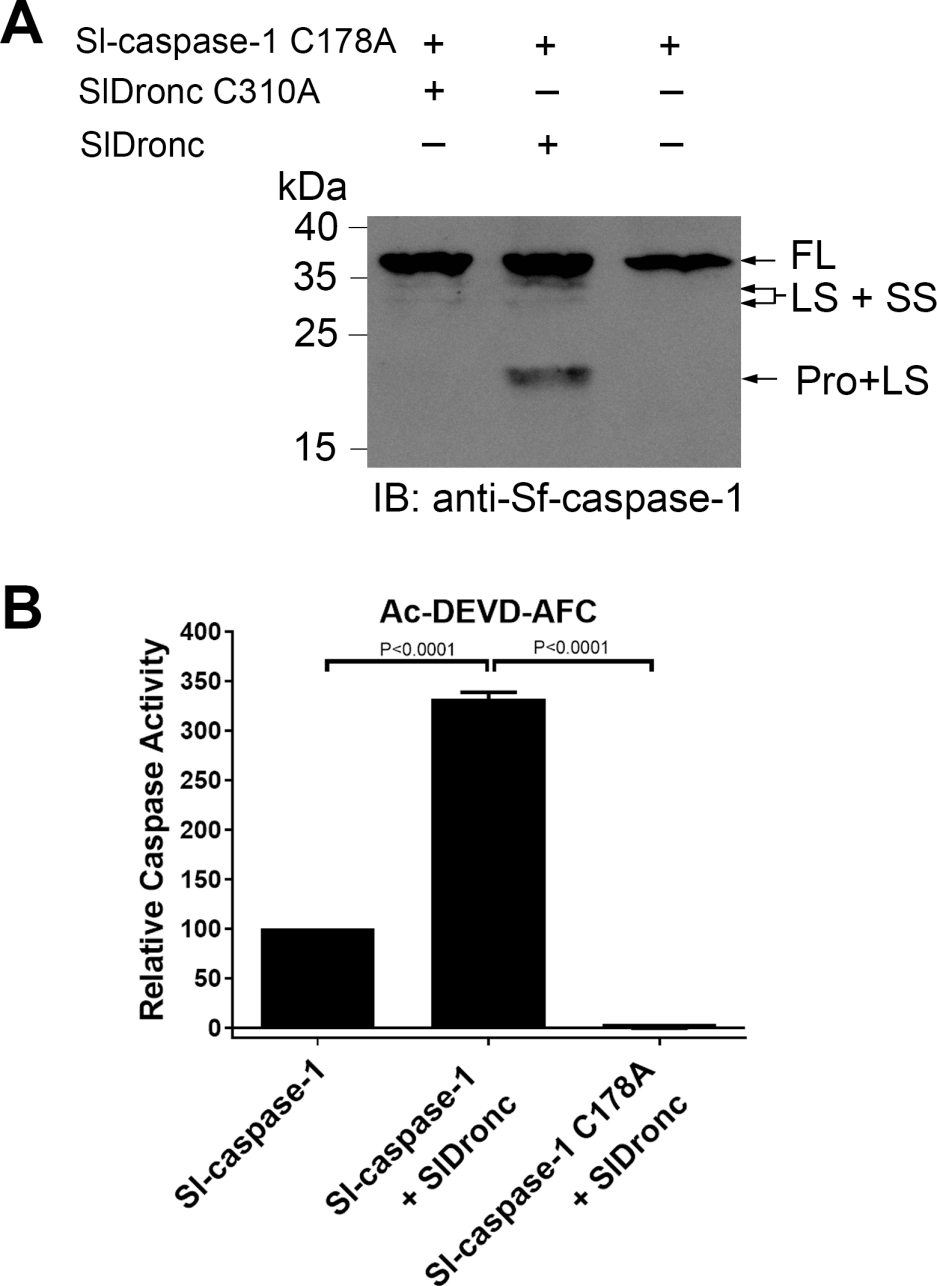
SlDronc’s in vitro cleavage and activation of Sl-caspase-1. (A) Sl-caspase-1 C178A (600 nM) was incubated respectively with 600 nM of SlDronc or SlDronc C310A with C-terminal His-tag at 37 ° C for 3 h in Na-Citrate buffer , and then the mixtures were detected by western blotting using Anti-Sf-caspase-1 antibody . (B) Sl-caspase-1 (5 nM) was incubated with buffer and SlDronc (240 nM) respectively. Sl-caspase-1 C178A (5 nM) was incubated with SlDronc (240 nM). Then caspase activity of the mixtures were measured using synthetic caspase substrate Ac-DEVD-AFC (20 µM). Caspase activity was indicated relative to that of Sl-caspase-1 (5 nM) or Sf-caspase-1 (5 nM) incubated with buffer. SD from three independent experiments were presented, and statistical significance was analysied by *t* test.

It is widely known that initiator caspase can cleave effector caspase to activate effector caspase. To determine whether SlDronc can cleave recombinant Sl-caspase-1 (an effector caspase in *S. littoralis*), we expressed and purified Sl-caspase-1 and Sl-caspase-1 C178A (a catalytic site mutant) in *E. coli*. To avoid autocatalytic cleavage of Sl-caspase-1, we used Sl-caspase-1 C178A to detect cleavage by SlDronc. Recombinant SlDronc was incubated with Sl-caspase-1 C178A at 37 °C for 3 h, and we analyzed the incubation mixture using western blotting and an Anti-Sf-caspase-1 antibody. The result showed that Sl-caspase-1 C178A was cleaved by SlDronc, but not by SlDronc C310A (Fig. 5A), suggesting that SlDronc could cleave Sl-caspase-1 directly *in vitro* and that SlDronc’s caspase activity was necessary. Additionally, we found that incubation with SlDronc significantly increased the enzymatic activity of Sl-caspase-1 on Ac-DEVD-AFC (Fig. 5B), further confirming that SlDronc can activate Sl-caspase-1. Therefore, we suggest that SlDronc may function as an initiator caspase in the *S. littoralis* apoptotic pathway.

### Overexpression of SlDronc-induced apoptosis in SL2 cells

**Figure 6:**
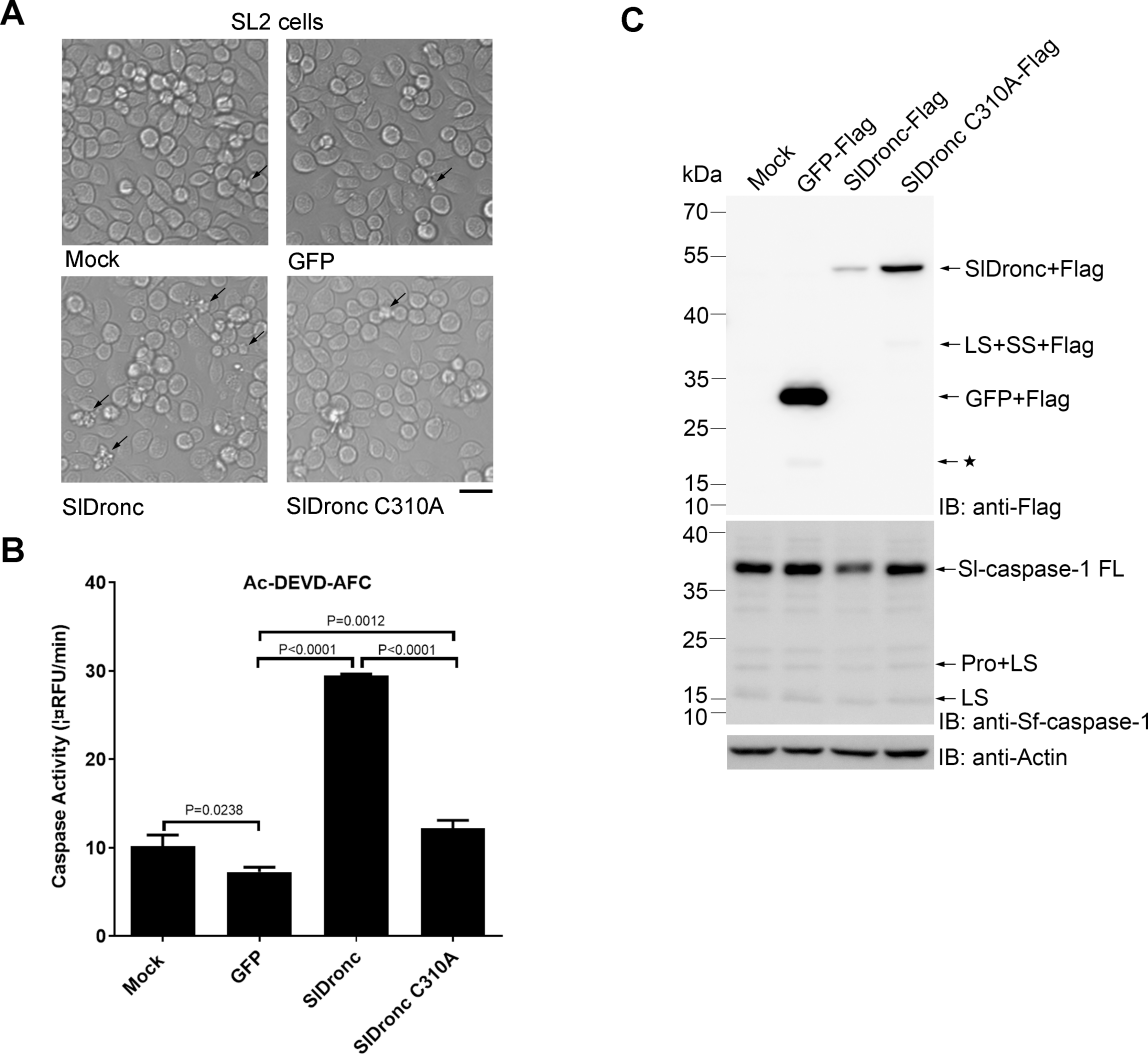
Overexpression of SlDronc-induced apoptosis in SL2 cells. SL2 cells were transfected separately with plasmids which express C-terminally Flag-tagged wild type SlDronc, catalytic site mutant SlDronc C310A and GFP for 24 h and subject to the following analysis. (A) SL2 cell pictures were taken (magnification ×200), the scale bar represents 50 µm. Arrows were used to mark the apoptotic cells. (B) and** (C)** SL2 cells were harvested and the prepared cell lysates were subjected to caspase activity assay (B) or western blotting utilizing antibodies against Flag-tag, Sf-caspase-1 or β-actin (C). ≪: could be product of initiating from inner ATG. Changes in RFU (relative fluorescence units) per minute was used to show caspase activity. SD from three independent experiments were presented, and statistical significance was analysied by *t* test.

To determine whether SlDronc functions as an apoptotic caspase, we transfected plasmids that either expressed wild type SlDronc or the catalytic site mutant SlDronc C310A with a C-terminal Flag-tag, into SL2 cells, and we observed the cells across multiple intervals. Untreated cells and cells transfected with plasmid that expressed C-terminally Flag-tagged GFP were used as controls. At 24 h post transfection, we observed apoptosis in SL2 cells that expressed SlDronc, only slight apoptosis in SL2 cells that expressed SlDronc C310A, and no apoptosis in mock-treated cells or cells that expressed GFP (Fig. 6A). Moreover, lysate prepared from the cells that transiently expressed SlDronc showed significantly increased caspase activity on Ac-DEVD-AFC when compared to cells that transiently expressed GFP (Fig. 6B), which is consistent with the morphological results described previously. Western blot analysis that used an antibody against Flag-tag showed that the full-length SlDronc’s protein level was significantly lower than that of SlDronc C310A, suggesting that wild type SlDronc is cleaved when transiently expressed in SL2 cells (Fig. 6C). Western blot analysis using an antibody against Sf-caspase-1 showed that the full-length Sl-caspase-1 protein level was lower in cells that overexpressed SlDronc compared to cells that overexpressed GFP (Fig. 6C). Taken together, these findings indicate that apoptosis in SL2 cells was induced by SlDronc overexpression, and that SlDronc caspase activity was required to induce apoptosis. Therefore, SlDronc may be an apoptotic initiator caspase in SL2 cells.

### *Sldronc* knockdown decreased apoptosis induced by UV irradiation in SL2 cells

**Figure 7:**
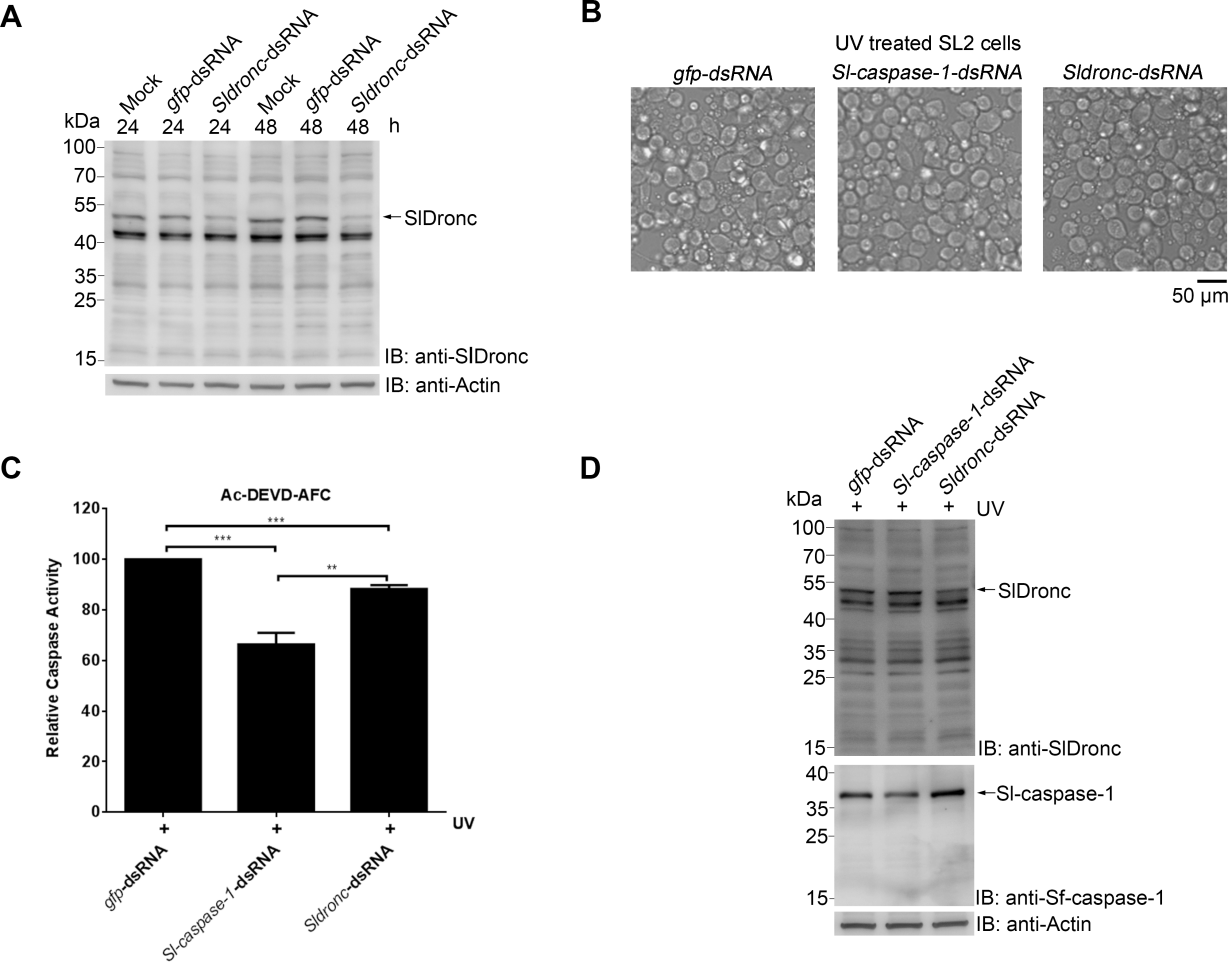
*Sldronc* knockdown decreased apoptosis induced by UV irradiation in SL2 cells. (A) SL2 cells were transfected with *Sldronc*-dsRNA or *gfp*-dsRNA. At 24 h and 48 h post transfection, cells were harvested and cell lysates were subjected to western blotting with antibody against SlDronc or β-actin. (B–D) SL2 cells were transfected with *Sldronc*-dsRNA,* gfp*-dsRNA or *Sl-caspase-1*-dsRNA for 24 h and then treated with UV irradiation. At 24 h post UV treatment, SL2 cells were subjected to following analysis. (B) SL2 cell pictures were taken (magnification ×200), and the scale bar represents 50 µm. (C) SL2 cells were harvested and caspase activity of the cell lysates were measured using Ac-DEVD-AFC. (D) The cell lysates were subjected to western blotting utilizing antibodies against SlDronc, Sf-caspase-1 or β-actin. Caspase activity was indicated relative to that of SL2 cells** transfected** with *gfp*-dsRNA and treated by UV. SD from three independent experiments were presented, and statistical significance was analysied by *t* test.

In order to verify SlDronc’s function during apoptosis, we used dsRNA to knock down *Sldronc* expression. First, we proved that the prepared dsRNA against *Sldronc* (*Sldronc*-dsRNA) could successfully knock down endogenous *Sldronc* expression 24 h and 48 h after transfection (Fig. 7A). Next, we transfected SL2 cells with *Sldronc*-dsRNA for 24 h, followed by UV irradiation. The SL2 cells transfected with dsRNA against *gfp* (*gfp*-dsRNA) and the cells transfected with dsRNA against *Sl-caspase-1* (*Sl-caspase-1*-dsRNA) were used as controls. We observed decreased apoptosis in cells transfected with *Sldronc*-dsRNA or *Sl-caspase-1*-dsRNA compared to cells transfected with *gfp*-dsRNA (Fig. 7B). We harvested the SL2 cells and analyzed the cell lysates using caspase activity assay and western blotting. Consistent with the morphologic results, the caspase activity assay showed decreased activity on Ac-DEVD-AFC in cells transfected with *Sldronc*-dsRNA or *Sl-caspase-1*-dsRNA when compared to cells transfected with *gfp*-dsRNA and treated with UV irradiation (Fig. 7C). The western blotting results showed that *Sldronc* knockdown significantly decreased Sl-caspase-1 cleavage induced by UV irradiation treatment in SL2 cells (Fig. 7D). These results confirmed SlDronc’s involvement in apoptosis induced by UV irradiation in SL2 cells.

### Increase in processed SlDronc forms in the presence of N-terminally truncated SlIAP

**Figure 8:**
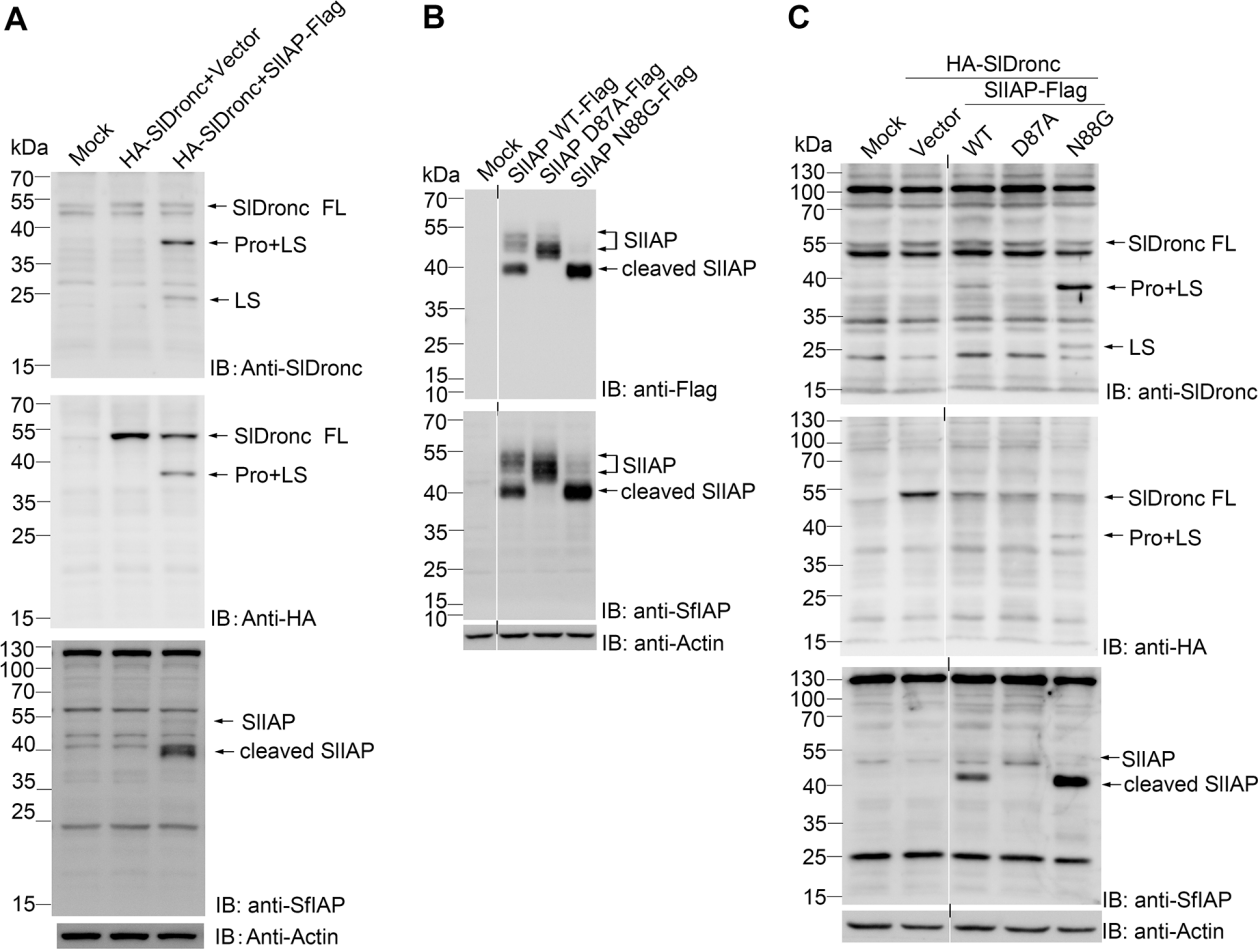
Increase in processed SlDronc forms in the presence of N-terminally truncated SlIAP. SL2 cells were transfected with plasmids respectively. Vector plasmid was used to make sure SL2 cells in each well were transfected with same amount of plasmid. At 24 h post transfection, cells were harvested and cell lysates were subjected to western blotting. (A) SL2 cells were transfected with plasmid expressing N-terminally HA-tagged SlDronc and plasmid expressing C-terminally Flag-tagged SlIAP, cells transfected with plasmid expressing SlDronc alone and cells mock treated were used as controls. (B) SL2 cells were transfected with plasmids expressing SlIAP WT, SlIAP D87A or SlIAP N88G respectively. (C) SL2 cells were cotransfected with plasmid expressing SlDronc and plasmid expressing SlIAP WT, SlIAP D87A or SlIAP N88G respectively, cells transfected with plasmid expressing SlDronc alone and cells mock treated were used as controls. A short vertical line indicates where lanes were removed and divided parts of the same western blot image were joined together.

IAP is vital in the regulation of apoptosis, and it is known to suppress caspase-mediated apoptosis in *D. melanogaster*, *B. mori,* and *S. frugiperda* (Hamajima et al., 2016; Kaiser, Vucic & Miller, 1998; Muro, Hay & Clem, 2002). To test the effects of SlIAP on SlDronc, we co-expressed SlIAP with SlDronc in SL2 cells. When SlDronc containing C-terminal Flag-tag was co-expressed with SlIAP, western blotting that utilized antibodies against Flag-tag only detected a decrease in full-length SlDronc, but not in processed SlDronc. In order to distinguish between Pro+LS (39 kDa) and LS+SS (36 kDa) detected by western blotting utilizing an anti-SlDronc antibody, we co-expressed N-terminally HA-tagged SlDronc with C-terminally Flag-tagged SlIAP in SL2 cells. Western blotting using an antibody against SlDronc showed that the processed SlDronc protein levels increased while the full-length SlDronc protein levels decreased when co-expressed with SlIAP. The processed form of 39 kDa corresponded to the SlDronc form composed of the prodomain and large subunit (Pro+LS), and the processed form of 24 kDa corresponded to the large subunit of SlDronc (LS) (Fig. 8A, first panel). Western blotting using an antibody against HA-tag also showed that the full-length SlDronc protein level decreased and the processed SlDronc protein levels increased when co-expressed with SlIAP (Fig. 8A, second panel). Western blotting using an antibody against SfIAP showed that SlIAP mainly existed as cleaved SlIAP (about 40 kDa) when co-expressed with SlDronc in SL2 cells (Fig. 8A, third panel).

To further determine which portion of SlIAP functioned on SlDronc, we replaced the predicted cleavage site D87 with Ala (A) to avoid N-terminally truncation, and replaced destabilizing degron N88 at the N-terminus of cleaved SlIAP with Gly (G), a stabilizing residue in the N-end rule degradation pathway. When expressed in SL2 cells, the D87A mutation blocked cleavage, indicated by the absence of cleaved SlIAP (40 kDa). N88G mutation stabilized the cleaved SlIAP (Fig. 8B), confirming that SlIAP was cleaved at the site D87 in the N-terminal and that the cleaved SlIAP was degraded by the N-end rule degradation pathway. When co-expressed with SlDronc in SL2 cells, the D87A mutant failed to increase the processed SlDronc protein levels, while the N88G mutant significantly increased the processed SlDronc protein levels (Fig. 8C). These results suggest that SlIAP was cleaved at aspartate residue D87 in the N-terminal, the truncated SlIAP was degraded by the N-end rule degradation pathway, and processed SlDronc forms increased in the presence of N-terminally truncated SlIAP.

### SlDronc inhibition by P49

**Figure 9:**
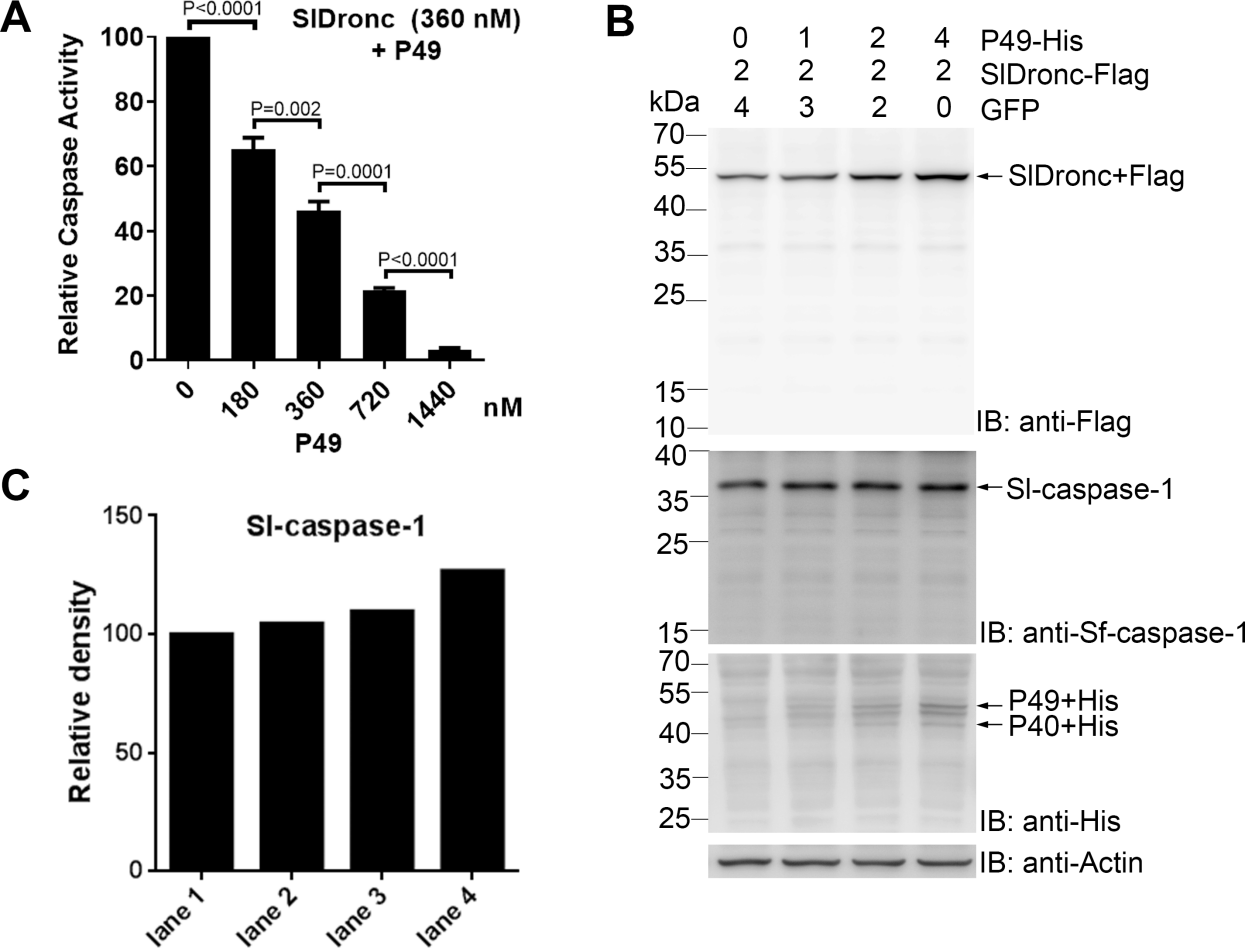
SlDronc inhibition by P49. (A) SlDronc (360 nM) was incubated with increasing amount of P49, and then the ability of the caspases to cleave Ac-VEID-AFC was determined. Caspase activity was indicated relative to that of SlDronc incubated with buffer. SD from three independent experiments were presented, and statistical significance was analysied by *t* test. (B) SlDronc was co-expressed with increasing amount of P49. Plasmid expressing GFP was used to make sure SL2 cells in each well were transfected with same amount of plasmid . At 24 h post transfection, SL2 cells were harvested and cell lysates were analysied by western blotting utilizing antibodies against Flag-tag, Sf-caspase-1, His-tag or β-actin. (C) Protein grayscale analysis of full length Sl-caspase-1 was performed using Quantity One. Band density was indicated relative to that of cells expressing SlDronc and GFP.

P49, a baculoviral apoptosis inhibitor in SpliNPV, inhibits caspase activity of initiator caspases. P35, a baculoviral apoptosis inhibitor in AcMNPV, inhibits the caspase activity of effector caspases (Jabbour et al., 2002; Zoog et al., 2002). To determine whether SlDronc was regulated by P49, we tested the caspase activities of recombinant SlDronc that had been expressed and purified in *E. coli* by incubating them with increasing amounts of P49. The results showed that P49 inhibited SlDronc caspase activity in a dose-dependent manner (Fig. 9A). Using a 1:1 P49:SlDronc ratio, we reduced SlDronc’s enzymatic activity by 54%. SlDronc activity was reduced by 97% when the ratio was 4:1. To further test P49′ s effect on SlDronc in SL2 cells, we co-expressed SlDronc with increasing amounts of P49. Western blot analysis showed that the full-length SlDronc protein level increased gradually with increasing amounts of P49 when co-expressed with SlDronc (Fig. 9B). Protein grayscale analysis of full-length Sl-caspase-1 showed that the protein levels also increased slightly (Fig. 9C). These data indicate that SlDronc was inhibited by P49.

## Discussion

Dronc has proved to be an essential initiator caspase as it cleaves and activates effector caspases to cleave downstream proteins in the apoptotic pathway, ultimately leading to apoptosis (Daish, Mills & Kumar, 2004; Dorstyn et al., 1999; Quinn et al., 2000). Dronc homologs from several Lepidopteran insects have been studied, including BmDronc, LdDronc, and SfDronc (Huang et al., 2013; Kitaguchi et al., 2013; Suganuma et al., 2011). In this study, we identified SlDronc, the first *S. littoralis* initiator caspase. SlDronc possesses typical initiator caspase features and was shown to induce apoptosis in SL2 cells.

Both mammalian and *Drosophila* caspases are subject to IAP regulation, and caspase ubiquitylation by IAPs can be either degradative or nondegradative (Darding & Meier, 2012; Muro, Hay & Clem, 2002; Vaux & Silke, 2005). In degradative ubiquitylation, caspases are degraded by proteasome. While nondegradative ubiquitylation does not lead to the degradation of caspases, it has been proposed that ubiquitin (Ub) can sterically occlude substrate entry and cause a conformational change in caspases by reducing their catalytic processivity (Ditzel et al., 2008). Several studies have shown that ubiquitylated, full-length Dronc mediated by DIAP1 can be initially degraded by proteasome (Chai et al., 2003; Muro, Hay & Clem, 2002), while other researchers have found that processed and activated Dronc in the Dark apoptosome is degraded in a DIAP1-dependent manner (Lee et al., 2011; Shapiro et al., 2008). Notably, both cBm-IAP1 depletion and overexpression can stabilize the cleaved form of Bm-Dronc, despite the opposite effect occurring during apoptosis in BM-N cells (Hamajima et al., 2016). In this study, SlIAP overexpression also increased the cleaved SlDronc protein levels (Pro+LS and LS) and decreased the full-length SlDronc protein levels (Fig. 8A). This decrease observed in full-length SlDronc may be due to elevated ubiquitylation by the overexpressed SlIAP, but the mechanism by which the cleaved SlDronc protein levels increased (Pro+LS and LS) requires further study.

Identifying and functionally characterizing SlDronc further clarifies *S. littoralis’* apoptosis pathway. After receiving the apoptotic signal, SlDronc functions as an initiator caspase that cleaves and activates the effector caspase Sl-caspase-1, Sl-caspase-1 cleaves several cellular substrates that serves as the executioner of apoptosis in SL2 cells. SlIAP functions as the last line of defense against apoptosis. The identification of SlDronc will facilitate further studies on *S. littoralis’* apoptosis mechanism and baculovirus infection-induced apoptosis.

## Conclusion

We identified that SlDronc, an initiator caspase in *S. littoralis*, can cleave and activate effector caspases. SlDronc overexpression induced apoptosis in SL2 cells, and *Sldronc* knockdown decreased apoptosis induced by UV irradiation. Our results indicate that SlDronc is an apoptotic initiator caspase in *S. littoralis*. Additionally, we found that processed forms of SlDronc increased in the presence of N-terminally truncated SlIAP and that SlDronc was inhibited by P49. This study will contribute to the elaboration of *S. littoralis’* apoptotic pathway and may facilitate future studies on baculovirus infection-induced apoptosis.

##  Supplemental Information

10.7717/peerj.10329/supp-1 Supplemental Information 1 Raw data of catalytic activity measurement and images of unprocessed Western Blot

## Additional Information and Declarations

### Data Availability

The following information was supplied regarding data availability: The raw measurements and western blot images are available in the Supplementary Files.
